# Laboratory Evaluations of Correction Equations with Multiple Choices for Seed Low-Cost Particle Sensing Devices in Sensor Networks

**DOI:** 10.3390/s20133661

**Published:** 2020-06-30

**Authors:** Wen-Cheng Vincent Wang, Shih-Chun Candice Lung, Chun Hu Liu, Chen-Kai Shui

**Affiliations:** 1 Research Center for Environmental Changes, Academia Sinica, Nangang, Taipei 115, Taiwan; phdzen@gate.sinica.edu.tw (W.-C.V.W.); lch0909@gate.sinica.edu.tw (C.H.L.); kyle@gate.sinica.edu.tw (C.-K.S.); 2 Department of Atmospheric Sciences, National Taiwan University, Taipei 106, Taiwan; 3 Institute of Environmental Health, National Taiwan University, Taipei 106, Taiwan

**Keywords:** air pollutant sensor, sensor evaluation, aerosol sensors, particle correction equation, chamber evaluation, interclass correlations

## Abstract

To tackle the challenge of the data accuracy issues of low-cost sensors (LCSs), the objective of this work was to obtain robust correction equations to convert LCS signals into data comparable to that of research-grade instruments using side-by-side comparisons. Limited sets of seed LCS devices, after laboratory evaluations, can be installed strategically in areas of interest without official monitoring stations to enable reading adjustments of other uncalibrated LCS devices to enhance the data quality of sensor networks. The robustness of these equations for LCS devices (AS-LUNG with PMS3003 sensor) under a hood and a chamber with two different burnt materials and before and after 1.5 years of field campaigns were evaluated. Correction equations with incense or mosquito coils burning inside a chamber with segmented regressions had a high R^2^ of 0.999, less than 6.0% variability in the slopes, and a mean RMSE of 1.18 µg/m^3^ for 0.1–200 µg/m^3^ of PM_2.5_, with a slightly higher RMSE for 0.1–400 µg/m^3^ compared to EDM-180. Similar results were obtained for PM_1_, with an upper limit of 200 µg/m^3^. Sensor signals drifted 19–24% after 1.5 years in the field. Practical recommendations are given to obtain equations for Federal-Equivalent-Method-comparable measurements considering variability and cost.

## 1. Introduction

Particulate matter with an aerodynamic diameter less than or equal to 2.5 μm (PM_2.5_) is a classified human carcinogen [1]. The Global Burden of Disease Study 2015 showed that around 5.7–7.3 million deaths could be attributable to PM_2.5_ exposure [2,3]. Many areas worldwide experience annual mean levels of PM_2.5_ reaching 100 μg/m^3^ [4,5], much higher than 10 μg/m^3^, the value recommended by the World Health Organization [6]. Since monitoring stations equipped with expensive instruments established by environmental regulatory agencies are only situated in limited areas, the development of low-cost sensors (LCSs) provides opportunities to measure pollutant levels at much higher spatial densities than ever before [7,8,9]. However, most LCSs for air pollutants face the data accuracy challenges [9,10], as they are typically not calibrated by the manufacturers due to cost considerations. Inaccurate underestimated pollutant levels may give false impressions of acceptable air quality, while inaccurate overestimated pollutant levels (2–3 fold, [10]) may mislead residents and result in unnecessary societal costs. Either way, biased LCS networks have limited applications.

In Taiwan, citizens have placed PM_2.5_ LCSs near their households or inside elementary schools due to concerns about the harmful effects of PM_2.5_. With the assistance of information scientists and a volunteer internet groups, since 2016 real-time PM_2.5_ values all over Taiwan have been made available on a website (https://v5.airmap.g0v.tw/#/map) to show the spatial distributions of PM_2.5_. The temporal trends and relative comparisons of LCS data among different areas have met the demand of citizens who want to be informed of the PM_2.5_ levels in their neighborhoods. Pollution awareness has thus been enhanced dramatically. However, overestimated PM_2.5_ levels from these LCSs often needlessly alarm citizens who are unaware of the aforementioned accuracy issue, and environmental groups have wrongly accused the Taiwan Environmental Protection Administration (Taiwan EPA) of tampering with the data of official monitoring stations, which show consistently lower levels than those of the LCSs (https://news.housefun.com.tw/news/article/154493172776.html). The unnecessary distrust between citizen groups and the Taiwan EPA is an unfortunate side effect of this successful collaboration between academics and citizens. Solving the data accuracy issue could also resolve the dilemma of the current PM_2.5_ LCS network in Taiwan and in other countries, as well as enhance the applicability of these environmental LCS networks.

Rai et al. [8] proposed a two-stage calibration process with laboratory calibration done by the manufacturers and calibration checks performed by the end-users. This would be ideal if the manufacturers followed the authors’ suggestions. However, demanding manufacturers to calibrate LCSs may be unrealistic since LCSs are made in larger quantities with much lower costs than more expensive instruments. The alternative is to obtain research-grade observations by conducting side-by-side comparisons and establishing correction equations to accordingly convert LCS readings into data comparable to those obtained from research-grade instruments, such as GRIMM, SidePak, and tapered element oscillating microbalance (TEOM) analyzers [8,10,11,12]. However, these evaluations are labor-intensive, time-consuming, and resource-demanding, which is not possible for the thousands of LCSs placed by citizens in large areas. Innovative ways to correct the data of these LCSs to nearly research-grade observations are thus urgently needed.

The alternative to correction depends on data science. A review paper [13] highlighted the growing use of machine learning and other advanced data processing approaches to improve LCS/monitoring agreements with reference monitors. One of the machine learning methods, random forest, has been applied to calibrate LCSs for CO, NO_2_, CO_2_, and O_3_ with meteorological data and net responses from all sensors [14]. In addition, in preliminary explorations, our group found that deviations of the LCS signals from reference instruments are greatly reduced by applying machine learning methods to correct uncalibrated LCS readings in sensor networks with data from research-grade instruments taken from regulatory monitoring stations within a 3 km radius; these results will be summarized in another manuscript [15]. In short, machine learning methods are promising for reducing LCS deviations in sensor networks. However, these applications are restricted to the currently limited number of official monitoring stations.

Here, we propose a hybrid method for combining traditional laboratory evaluations and new data science methods to adjust LCS readings to research-grade observations. For LCS sets located within a 3 km radius of regulatory monitoring stations, machine learning could be applied to adjust LCS readings based on the monitoring instruments. For other LCSs in areas without monitoring stations, “seed” LCSs corrected with side-by-side comparisons in the laboratory could be installed strategically in those areas to provide research-grade observations to further correct nearby uncalibrated LCSs. This combination of traditional laboratory evaluations and new machine learning methods could largely enhance the scientific and social values of such sensor networks. As a result, environmental scientists with the ability to conduct laboratory evaluations could further contribute to community monitoring and citizen science by improving the data accuracy of these LCS networks. This paper is focused on acquiring research-grade data for the seed LCS. The second part involves applying machine learning methods and will be presented in another manuscript [15].

Laboratory evaluations have been conducted for several PM LCSs, such as those from Alphasense, Dylos, Samyoung, Sharp, Shinyei, Nova, and Plantower. Rai et al. [8] reviewed evaluations published before 2017. Subsequently, at least seven publications have presented results from laboratory evaluations for various PM LCSs [16,17,18,19,20,21,22]. Among the sensors evaluated, Plantower sensors, which have a relatively low cost (~35 USD), consistently performed well in terms of the intra-precision among themselves and their precision compared to various research-grade instruments [16,17,18,19,20,21,22]. It was found that Plantower sensors performed better than Shinyei ones because Plantower sensors are designed with a fan that draws in air and a laser light source [23]. Moreover, there are more than 4000 PMS sensors in the PM sensor network of Taiwan. Thus, a Plantower PMS3003 sensor was chosen as the target LCS in this work for the reasons stated in the Section 2. To date, only five publications have focused on PMS3003 [10,16,23,24,25]. Therefore, new laboratory evaluation results can fill the data gap for this LCS and facilitate its application in environmental studies.

With a hybrid method of data correction in mind, it is important to provide valid and robust laboratory correction equations for limited “seed” devices with LCSs (i.e., AS-LUNGs, Academia Sinica, Taipei, Taiwan). In addition, “low-cost” is an important consideration for evaluating these LCS devices to facilitate their wide application. The objective of the current work is to obtain reliable and robust correction equations to convert LCS signals to research-grade data with side-by-side comparisons between research instruments and LCS devices. The robustness of these equations was evaluated under two different experimental settings with two different burnt materials and both before and after 1.5 years of field campaigns. Correction equations in different concentration ranges were also established, and possible ceiling values were explored. Recommendations are given for evaluation methodologies to be applied by other research groups with consideration of different financial requirements and different degrees of variability. This work shows two candidates can be used as “seed” LCS devices. LCS readings can be converted to data comparable to the Federal Equivalent Method (FEM) based on side-by-side comparisons in a laboratory. For scientists with resources, evaluation experiments can be conducted with incense burning in a customer-built chamber with FEM instruments to obtain correction equations with coefficients of determination (R^2^) of 0.999, less than 6.0% variability for PM_2.5_ and PM_1_ in slopes, and mean root-mean-square-errors (RMSEs) of 1.18 and 1.56 µg/m^3^ for PM_2.5_ and PM_1_, respectively, in a range of 0.1–200 µg/m^3^. For scientists with limited resources, experiments can be conducted using a standard chemical fume hood with an R^2^ of 0.930–996, less than 15.5% variability in the slopes, a mean RMSE of 2.4 for PM_2.5_, and 10.1% variability in the slopes with a mean RMSE of 1.82 for PM_1_.

## 2. Materials and Methods

### 2.1. Sensors and Instruments

PMS3003 (Plantower, Beijing, China) sensors were chosen based on our previous collaboration with information scientists that found PMS3003 to have good precision, with an R^2^ between PMS3003 and a GRIMM sensor results as high as 0.983 for PM_1_ and 0.984 for PM_2.5_ [16]. This sensor has high precision, but its bias needs to be corrected to acquire accurate data. PMS3003 has a laser light source, and 90° scattered light is detected by a photo-diode detector with a laser wavelength (650 ± 10 nm) close to that of GRIMM 1.109 (655 nm) [23]. It can detect particles greater than ~0.3 µm [8]. Based on our experience, this sensor’s mean time to failure is longer than 3 years, which is close to the time to failure claimed by the manufacturer [20]. PMS3003 with a volume-scattering detection approach can obtain PM measurements that are independent of the flow rate [24]. Field evaluations in Taiwan also showed that PMS3003 provides stable readings in ambient monitoring, indicating that its performance is not interfered with by high relative humidity (RH%, 74 ± 11%) [10]. Although PMS3003 is not the newest Plantower sensor, it has potential to be used as a “seed” LCS high due to its precision, stability, and long lifetime.

The PMS3003 needs to be integrated with power and data transmission components to become an LCS device for use in the relevant applications. The LCS devices with PMS3003 evaluated in this work were the AS-LUNG-P and AS-LUNG-O, as integrated by our team (AS stands for Academia Sinica, the research institute supporting its development, LUNG indicates the human organ most commonly affected by air pollutants, and O and P indicate “outdoor” and “portable” versions, respectively). The AS-LUNG-P (~270 USD basic manufacturing cost) is 135 mm × 70 mm × 40 mm in size and 153 g in weight and must be connected to a mobile battery or electric socket as a power supply [26]. It can be installed in the open air under a rain cover to monitor ambient PM. For the AS-LUNG-O (~650 USD basic manufacturing cost), the sensors are placed in a waterproof shelter connected to a solar panel with backup batteries for the power supply, with the option of using household electricity where easily accessible. The size of the whole set is roughly 60 cm (W) × 50 cm (D) × 50 cm (H), with a weight of approximately 4.8 kg. Data from AS-LUNG-P and AS-LUNG-O can be transmitted wirelessly by the built-in WiFi or 4G modules to a cloud database. An SD card was added as a complement to avoid data loss during wireless transmission. The data correction of AS-LUNG-O with GRIMM and its application for community source evaluations were presented in a previous study [10]. In this work, AS-LUNG-P and AS-LUNG-O, with 15 s and 1 min resolutions, respectively, for both PM_2.5_ and PM_1_ readings, were compared against research-grade instruments in side-by-side laboratory evaluations. The PM correction equations were established based on 1 min averages.

The research-grade instrument used for the side-by-side comparison was a GRIMM 1.109 (GRIMM Aerosol Technik Ainring GmbH & Co, Ainring, German), which is an aerosol spectrometer that detects aerosols in a size range of 0.25–32 μm in 31 size channels. The flow rate is 1.2 L/min with a limit of detection of 0.1 μg/m^3^ and reproducibility of 5%. During the side-by-side evaluation of AS-LUNG in a hood or chamber, as described in Section 2.2, the sampling interval was set to 1 min.

**Figure 1:**
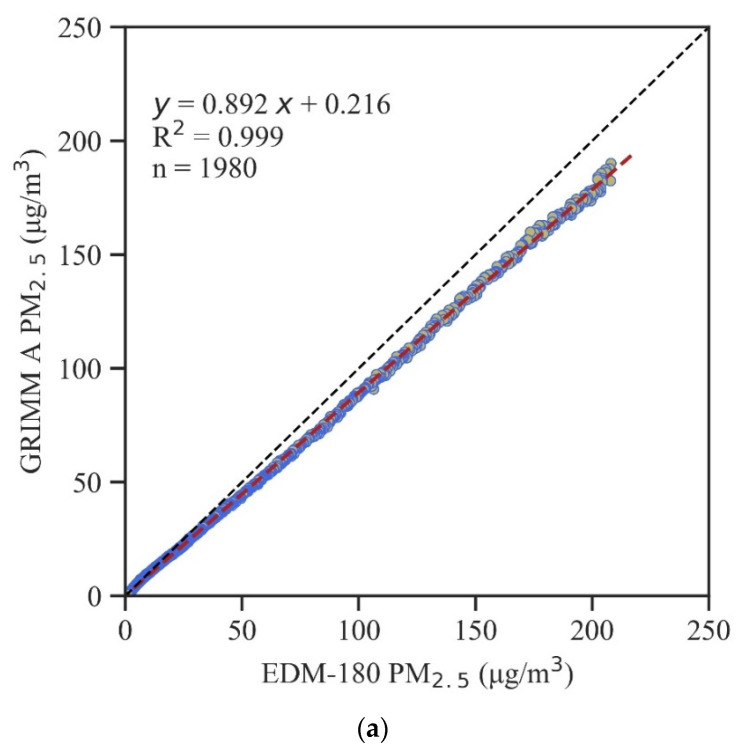

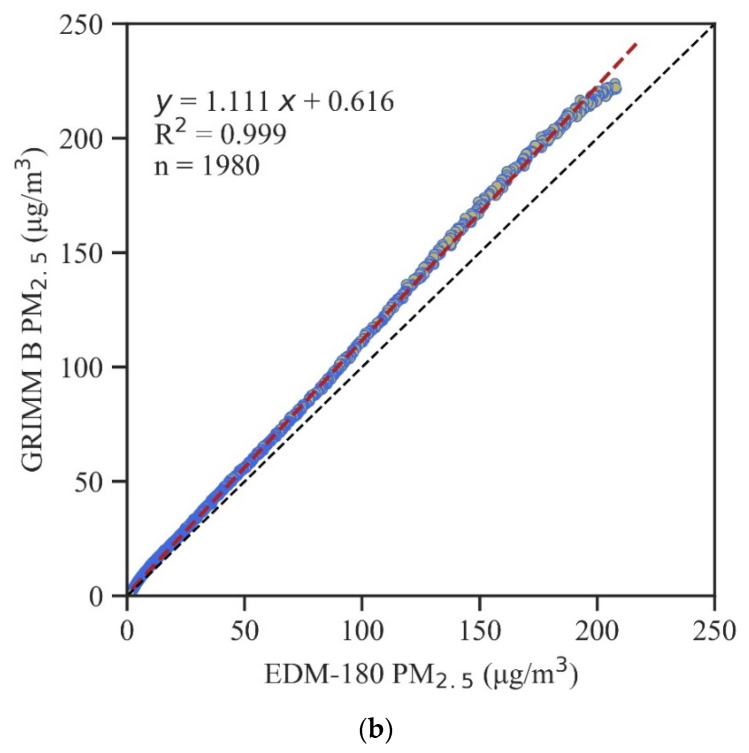
Comparisons for PM_2.5_ with EDM-180 and (**a**) the first GRIMM 1.109 (GRIMM A) under a temperature of 26.2–29.0 °C and humidity of 46–67%; (**b**) the second GRIMM 1.109 (GRIMM B) under a temperature of 25.0–29.0 °C and humidity of 50–75%.

In addition, the two GRIMM instruments used in the AS-LUNG laboratory comparisons (GRIMM A and B) were compared against an EDM-180 (GRIMM Aerosol Technik Ainring GmbH & Co, Ainring, Germany), a Federal Equivalent Method (FEM) instrument designated by the United States Environmental Protection Administration (USEPA) for PM_2.5_, which uses the light-scattering principle. A side-by-side comparison of GRIMM and DEM-180 was conducted in the chamber described in Section 2.2 with incense smoke used as the test material. The data agreement was quite good, with an R^2^ = 0.999 and a bias of about 11% for PM_2.5_ and PM_1._ Figure 1 shows a comparison between the results of the EDM-180 and the two GRIMM instruments for PM_2.5_ under the specified conditions. In order to provide common ground to compare the evaluation results across the AS-LUNG sets with either GRIMM A or GRIMM B, correction equations for converting AS-LUNG readings to EDM-180 comparable values are presented in this paper.

### 2.2. Hood and Chamber

**Figure 2:**
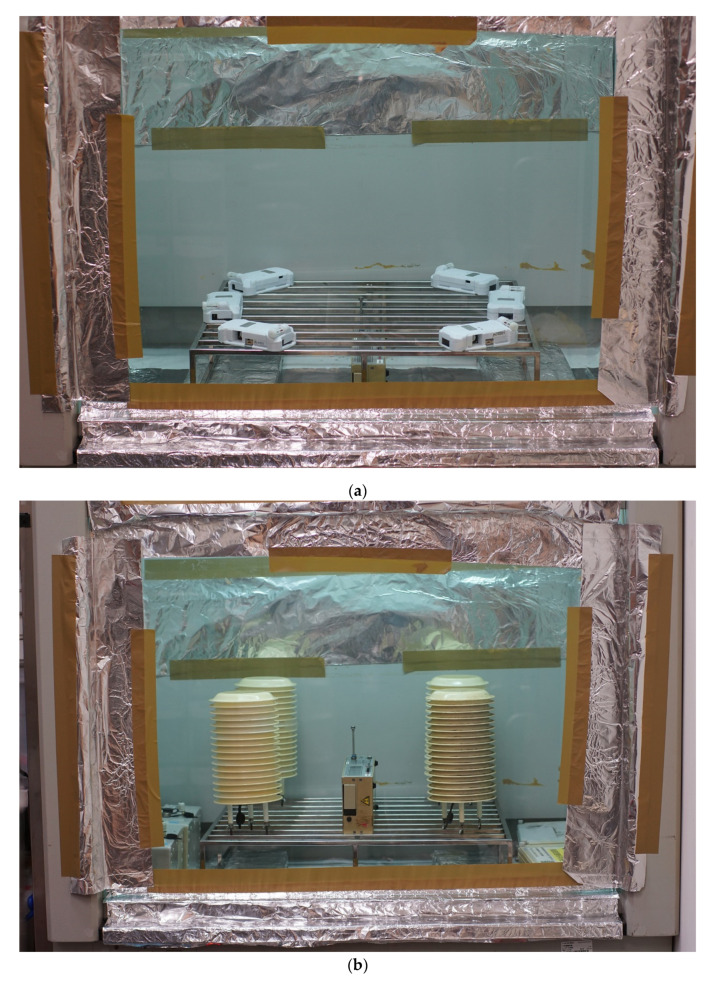

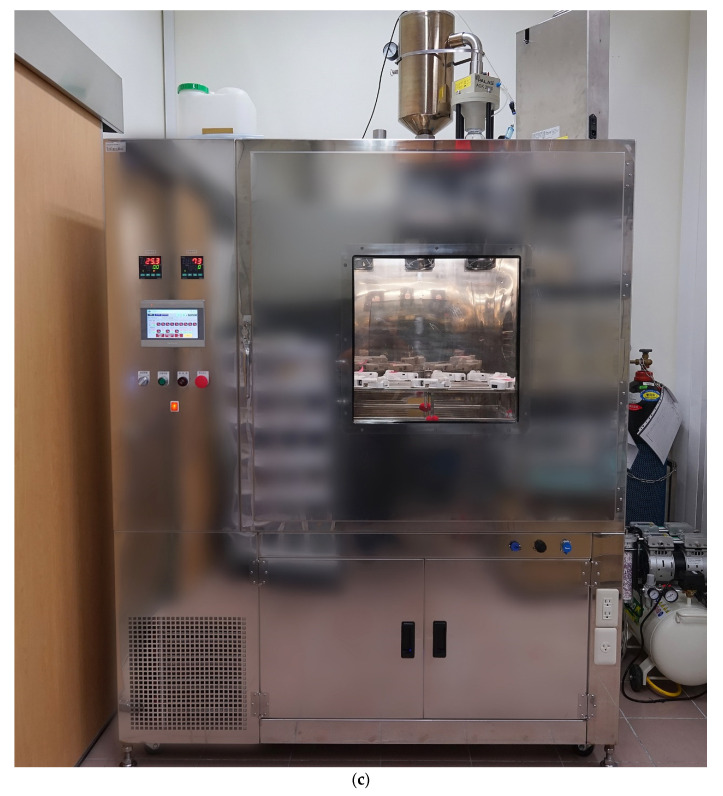
Side-by-side comparisons inside (**a**) a hood with 6 AS-LUNG-P sets, (**b**) a hood with 4 AS-LUNG-O sets, and (**c**) a chamber with 9 AS-LUNG-P sets.

Side-by-side comparisons of the GRIMM and AS-LUNG sensors were conducted under two different experimental settings inside an almost closed chemical fume hood and an enclosed chamber. The procedures for the collocated experiments inside the chemical fume hood are described in [10]. Briefly, the AS-LUNG sensor sets were evaluated in batches against one GRIMM instrument inside a chemical fume hood (100 cm × 60 cm × 115 cm, Figure 2a,b). An incense stick was ignited in the center on the hood and burnt for 60–70 min. After burning ceased, the GRIMM and AS-LUNG readings in the decay periods were monitored for comparison. To avoid disturbing the internal conditions, the front of the hood was completely closed, and the sides were sealed with tape. The ventilation system of the hood was also turned off during the entire burning/decay period. Nevertheless, outside air could still seep in to support incense burning, while hot air inside could leak out from the top duct. Under slow air movement, the generated PM_2.5_ filled the hood and gradually vented from the top. Tests were conducted to ensure the homogeneity of the PM_2.5_ levels within the hood during the decay phase. Since the hood was not sealed tightly, the concentration decay occurred rapidly. Data were collected starting 10 min after the end of the incense-burning and lasted about 12–14 h. As a result, the sample sizes at relatively high levels (50–200 µg/m^3^) were much smaller than those in a lower range. Potential bias could be introduced with limited observations under high levels, thereby significantly affecting the slopes of the correction equations. 

Figure 2a,b show examples of the six AS-LUNG-P sets and four AS-LUNG-O sets evaluated in the hood experiments. To obtain correction equations suitable for actual field conditions, the sensors were evaluated with an outer case or waterproof shelter. In this work, the results of nine AS-LUNG-P sets and 12 AS-LUNG-O sets are reported. In addition, a duplicate experiment was conducted for each of these AS-LUNG sets to assess the reproducibility of the correction equations in the hood experiments.

To improve the not-so-well sealed conditions, side-by-side comparisons were conducted inside a newly constructed enclosed steel chamber (Figure 2c, ~33000 USD, Beta Science Co., Ltd., Taipei, Taiwan), which was custom-built for the purpose of evaluating the LCS devices with research-grade instruments. The inside of the chamber is 1 m × 1 m × 1 m with surface coatings to avoid the static effects of particles attached to the wall. There are six sampling ports located in the chamber. Five small fans (12 cm in diameter) were installed, with three in the back and two in the corner of the chamber which were turned on for 20 min at the beginning of the experiments to ensure well-mixed conditions.

In the chamber evaluation, the AS-LUNG sets were evaluated in batches against one GRIMM instrument with an incense stick ignited and burnt for 230 s to generate PM_2.5_ concentrations roughly higher than 200 μg/m^3^ (GRIMM measurements) inside the chamber. After 230 s, the incense stick was taken out quickly, and the chamber was closed tightly except for the bottom port. A filter was connected to the bottom port since the pressure inside the chamber sometimes needs an outlet. The PM generated inside can be captured in this filter in case the pressured air vents out. The experiments were stopped after 48 h. The data used for the instrument comparisons were those after the maximum PM_2.5_ concentrations. Again, a duplicate experiment was conducted for each of the nine AS-LUNG-P sets to assess reproducibility. In the “high-level” experiments, the procedures were the same, except the incense sticks were burnt for 800 s to generate concentrations above 400 μg/m^3^ inside the chamber.

To facilitate the application of LCS devices under real environmental conditions, it is intended to conduct experiments under temperature and relative humidity (RH) conditions typical in Taiwan. The concerning impacts of RH on aerosol sensing are discussed in [10]. Briefly, there is an intrinsic difference in the PM_2.5_ concentrations obtained using sensors based on light scattering principles and those measured by filter-weighing sampling devices. Water droplets suspended in the air are, by definition, aerosols. USEPA has specified controlling filter weighing within 30–40% RH for regulatory purpose [27] to evaluate whether an area complies with the air quality standards. Nevertheless, LCS devices can serve purposes other than regulatory such as community monitoring and citizen science [28]. Actually, we argue against the need for humidity control in health-oriented studies because inhaled PM_2.5_ contains water droplets, which should be taken into account due to their health impacts. As the PM_2.5_ LCS devices in our research were not intended for regulatory purposes but instead aim to ease the concern of citizens regarding high pollutant levels that are harmful for public health, we did not control for humidity in our experiments. We specify the actual temperature and humidity conditions in the laboratory and field evaluations in the Results section.

### 2.3. Burnt Materials

Since these evaluations are focused on LCS devices, a low enough cost for evaluations is also of concern to decrease the burden of conducting LCS-related scientific studies. Thus, the burnt materials used for evaluation were purposely chosen based on combustible materials used frequently in Taiwan and around the world, rather than the expensive standard dust from the National Institute of Standards and Technology. Current worldwide health concerns are mainly focused on PM_2.5_ rather than PM_10_; thus, the present choice of combustible material (which mainly generates PM_2.5_) was ideal to meet our needs. Since most high PM_2.5_ occurs in high-density urban areas, especially in Asia, and different aerosols with different physio–chemical and light-scattering properties, we would like to choose test aerosols with similar light-scattering properties to urban PM_2.5_. Most primary urban PM_2.5_ particles are generated by combustion sources from industry, traffic, cooking, etc. Eight different PM sources were previously tested using another sensor from the same manufacturer, a PMSA003 [22]. Among these sources, incense, cooking, and residential air exhibited the highest accuracies and precisions. The authors concluded that the sensors could handle more real-world scenarios with high degrees of accuracy. The authors did not specify the cooking fuel used for their experiments, and residential air from Baltimore (MD, USA) was difficult to reproduce in other areas. Since the evaluation results for incense showed similar performance to the PM_2.5_ in residential air, suggesting incense may be a suitable substitute for urban PM_2.5_.

Therefore, incense sticks were chosen in this work considering the aforementioned reasons plus incense-burning is prevalent in Asia for religious purposes and around the world due to its fragrant smell and ability to aid relaxation. The emission factors of PM_2.5_ were previously evaluated in [29]. Additionally, our earlier work showed that the AS-LUNG-O data collected in the field corrected by laboratory correction equations (based on incense-burning evaluations) had only a 10 ± 9% absolute difference with collocated GRIMM sensors in the field, with a correlation of 0.93 ± 0.05 [10]. This demonstrated that the correction equations obtained with incense-burning were applicable to the field in Taiwan. The incense sticks used were purchased from local Taiwan markets (Hong, Shun-Li commercial firm, New Taipei, Taiwan). These sticks are made from sandalwood and contain several natural fragrances. Each whole stick was 39.5 cm in length; the combustible part was 2.2 mm in diameter and 29.0 cm in length.

The potential biases in the correction equations for the different burnt materials were also investigated with another burnt material. To avoid a fire hazard, there are limited choices of test materials available. A mosquito coil was chosen as another test material since it is also used frequently in Taiwan and widely around tropical and subtropical areas. Mosquito coils were purchased from a Taiwan market (Chung Tai Sing Chemical Industry Co., Ltd., Hsinchu, Taiwan) and burnt exactly the same way as the incense sticks. The diameter of each whole coil was 10.0 cm with a cross sectional area of 6.8 mm × 4.3 mm.

### 2.4. Sensor Drift

It is always a concern whether the sensor signals will drift over time. Thus, side-by-side comparisons of AS-LUNG-O with GRIMM in hood experiments before and after field monitoring were conducted to assess potential sensor drift. PM monitoring was conducted starting in July 2017 in a community surrounded by mountains in central Taiwan with eleven sets of AS-LUNG-O. After field operations for nearly 1.5 years, three AS-LUNG-O sets were taken back in early January 2019 for re-evaluation under the same laboratory setup with GRIMM to assess sensor drift (CONC_Post-Test_) and compared with the evaluations conducted before field monitoring (CONC_Pre-Test_). Of these three sets, two were cleaned once on-site, while one was not cleaned at all. The cleaning procedures included taking apart the components to remove dust, dead bugs, and spider webs. Each sensor was also cleaned with cotton swabs. Whether this cleaning affected the sensor drift was evaluated by comparing the sensor drift between the devices with and without cleaning.

### 2.5. Data Analysis

Regression equations were established based on 1 min observations. GRIMM measurements were converted to EDM-180 comparable data based on Figure 1a,b, which were then compared with the AS-LUNG readings to construct regression models, with EDM-180 as an independent variable and AS-LUNG as the dependent variable. These equations were compared with the slopes, R^2^, and RMSE. Accuracy was evaluated by comparing the slopes of these regression lines with the USEPA’s criteria (1 ± 1) [30]. Precision was evaluated against the USEPA’s criteria for continuous PM_2.5_ monitors (r > 0.9 or R^2^ > 0.81) [30] and for candidate equivalent methods (r > 0.97 or R^2^ > 0.94) [31]. Additionally, inter-sensor variability was assessed with the percent coefficients of variance (%CV = standard deviation/mean) of the slopes and the RMSE of different AS-LUNG sets under the same experimental settings with the same materials. The %CV was compared with the USEPA’s acceptable measurement uncertainty for continuous PM_2.5_ monitors (%CV < 10%) [30]. The intraclass correlation coefficient (ICC), a widely used reliability index in test–retest analyses with a value of 0–1, was calculated to examine the repeatability of the duplicate experiments [20,32]. A high ICC index indicates better repeatability. For before-and-after field monitoring (mentioned in Section 2.4), the absolute percentage difference was calculated as the absolute value of (CONC_Post-Test_ − CONC_Pre-Test_)/ CONC_Pre-Test_ (%) to assess signal drift.

A breakpoint seems to have occurred around 30 µg/m^3^ in the PM_2.5_ data from the chamber experiments. Therefore, both linear regressions and segmented regressions for a range of 0.1–200 µg/m^3^ were established for the chamber experiments. Segmented regressions were obtained based on the methods presented in earlier publications [33,34]. Further, two “high-level” experiments were conducted in the chamber for high concentrations above 400 µg/m^3^ to obtain correction equations in that range and search for the possible upper limits (ceiling values) of the PMS3003 sensors since such high levels do occur in some countries [35,36]. For chamber experiments, only the results of the AS-LUNG-P sets are provided to focus our discussions. The concentration ranges of 200 or 400 µg/m^3^ were determined by EDM-180 comparable values.

## 3. Results

### 3.1. Hood Versus Chamber Experiments

Laboratory evaluations were conducted with nine AS-LUNG-P sets (A1–A9) and 12 AS-LUNG-O sets (B1–B12) with duplicate experiments in the hood and chamber settings. To focus our discussion, only the slope, R^2^, RMSE, and sample size (*n*) from the first batch of these experiments are presented (Table 1). The temperature and humidity conditions are reported in the second row, and the inter-sensor variability assessed by the %CV of these results is presented in the last row.

For PM_2.5_, the slopes of the correction equations for AS-LUNG-P are 2.44–3.59 with intercepts of −2.49 to −10.1 from the hood experiments using incense and slopes of 2.80–3.10 with intercepts of −15.4 to −9.11 from the chamber experiments (Table 1a). Both results indicate that PMS3003 overestimates PM_2.5_ by roughly 2–3 fold. Likewise, PMS3003 roughly overestimates PM_1_ by about 1.4–2.2 fold (Table 1b). For the 12 AS-LUNG-O sets (B1–B12), the PM_2.5_ values are overestimated by about 2.1–2.9 fold and PM_1_ by about 1.5–2.0 fold (Table 1c). 

The AS-LUNG-O sets seem to provide less overestimation than the AS-LUNG-P sets. The waterproof shelter may account for part of this discrepancy. Nevertheless, the overestimation of PMS3003 is apparent.

The high R^2^ values in both the hood and chamber experiments show the high correlation and precision of the PMS3003 with EDM-180. For the AS-LUNG-P sets, the PM_2.5_ comparisons in the chamber experiments with better-sealed conditions consistently provided a high R^2^ greater than 0.991 compared to hood experiments, with an R^2^ of 0.930–0.996. These high R^2^ values demonstrate that the AS-LUNG readings can be converted to EDM-180 comparable measurements using the correction equations with excellent agreement. However, the most significant problem lies in the large intercepts in both the hood and chamber experiments, which make these LCS devices unworkable in areas with PM_2.5_ levels lower than 5–10 µg/m^3^, which is roughly the ratio of the intercept to the slope when the LCS reading is zero.

**Figure 3:**
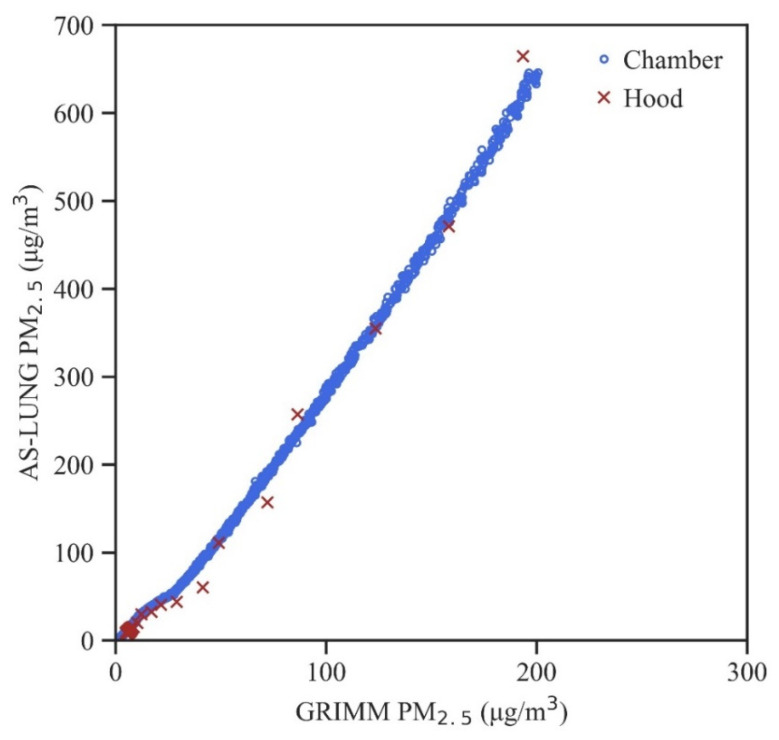
An example of the raw PM_2.5_ data obtained in the hood and chamber experiments with incense.

The mean RMSEs in the hood experiments for PM_2.5_ (1.67) and PM_1_ (1.38) are both smaller than the corresponding RMSEs (3.92 and 3.62, respectively) in the chamber experiments; this is because little data were in the 50–200 µg/m^3^ range in the hood experiment. Under the better-sealed conditions in the chamber experiments, a longer time was observed for the concentration decay; thus, observations with larger sample sizes were obtained under higher concentration ranges, compared to those in the hood experiments. Figure 3 shows an example of observations with sample sizes of 834, 3, and 2 at 1 min resolutions in the range of 0–50, 50–100, and 100–200 µg/m^3^, respectively, as measured in the hood experiment. More data at high levels are from the chamber experiments with sample sizes of 1104, 262, and 298, respectively. More observations at higher levels could enhance the robustness of these correction equations.

In terms of inter-sensor variability, the %CV of the slopes and the RMSEs for PM_2.5_ and PM_1_ from the chamber are both lower than the corresponding values from the hood for AS-LUNG-P, showing that the inter-sensor variability is greatly reduced in better-sealed environments. For AS-LUNG-O, the %CV of the slopes and the RMSEs for PM_2.5_ and PM_1_ are less than the corresponding %CV of AS-LUNG-P in the hood experiments. Again, the waterproof shelter may account for the lower variability.

For duplicate experiments, only the ICC indexes, compared to the first batch, are presented (Table 2). The ICCs for PM_2.5_ and PM_1_ for both the hood and chamber experiments using AS-LUNG-P are all high (0.928–0.999) (Table 2a), showing great repeatability among these experiments. The same was found for AS-LUNG-O in the hood experiments (Table 2b).

### 3.2. Linear versus Segmented Regressions

**Figure 4:**
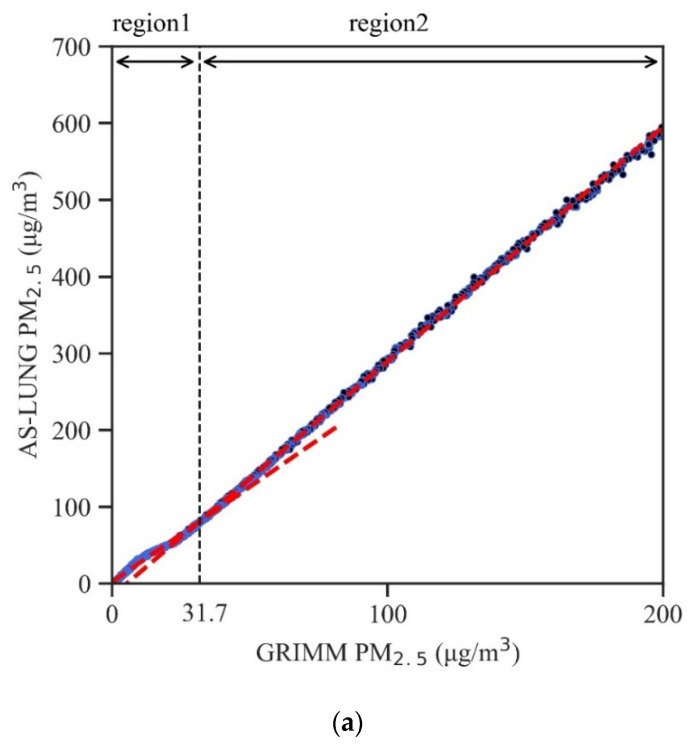

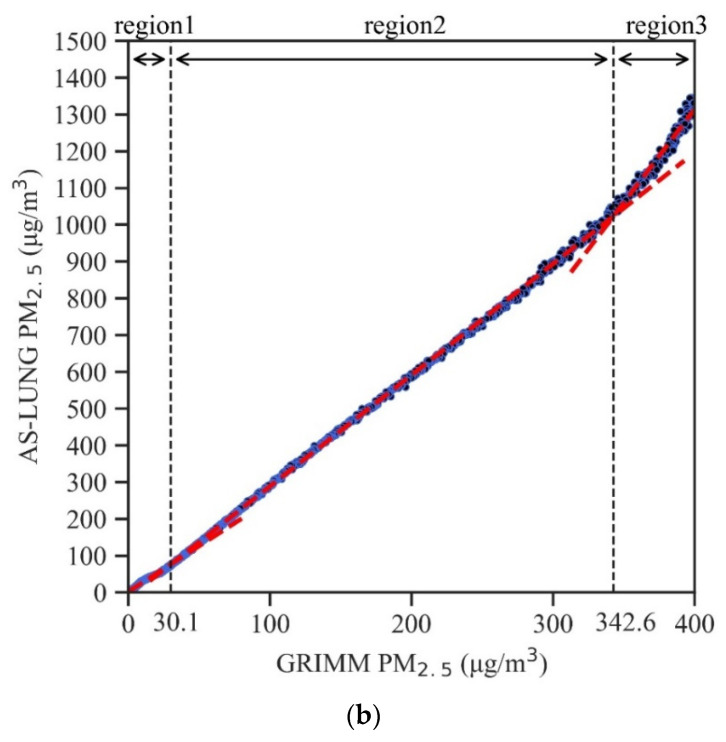
An example of raw PM_2.5_ data in the chamber experiments with incense with segmented regressions for (**a**) 0.1–200 µg/m^3^ and (**b**) 0.1–400 µg/m^3^; the regions indicated are the concentration ranges for the different regression equations.

There seems to be a breakpoint in PM_2.5_ in the chamber experiments at around 30–40 µg/m^3^ (Figure 4a). Therefore, segmented regressions were established and compared with the linear regressions (0.1–200 µg/m^3^) in the chamber experiments with incense for both PM_2.5_ and PM_1_ (Table 3a,b). These segmented regression equations have an excellent R^2^ (0.999) with an overall RMSE of 1.18 ± 0.07 µg/m^3^ for PM_2.5_ and 1.56 ± 0.15 µg/m^3^ for PM_1_ (Table 4a,b). In addition, the intercepts (the intercept in the first range, intercept 1) are much smaller in magnitude (0.46–2.24 µg/m^3^) than those in the linear regressions, allowing these LCS devices to be applicable in low-level areas. The %CV of the slopes and overall RMSEs are both less than 6% for PM_2.5_ and less than 11.8% for PM_1_, showing low inter-sensor variability. The RMSEs in the region with lower levels (region 1) are typically lower than those in the region with higher levels (region 2). Figure 4a shows an example of segmented regression in the range of 0.1–200 µg/m^3^ with two slopes indicated in the graph.

Moreover, segmented regressions were applied in the high-level experiments with PM_2.5_ levels up to 400 µg/m^3^. Table 3c,d present the regressions for ranges of 0.1–300 and 0.1–400 µg/m^3^, respectively. These equations have an excellent R^2^ (0.999), small intercepts (within absolute values of 2.2), and low inter-sensor variability (less than 9.9% for slopes and RMSEs). The mean RMSEs are 1.49 ± 0.11 µg/m^3^ for 0.1–300 µg/m^3^ and 1.77 ± 0.09 µg/m^3^ for 0.1–400 µg/m^3^ (Table 4c,d). Thus, for areas with high PM_2.5_ levels, segmented regressions up to 0.1–400 µg/m^3^ could be applied to obtain correction equations. Figure 4b shows an example of high-level segmented regressions with two breakpoints and three regressions.

### 3.3. High-Level Curves and Ceiling Values for PM_1_

**Figure 5:**
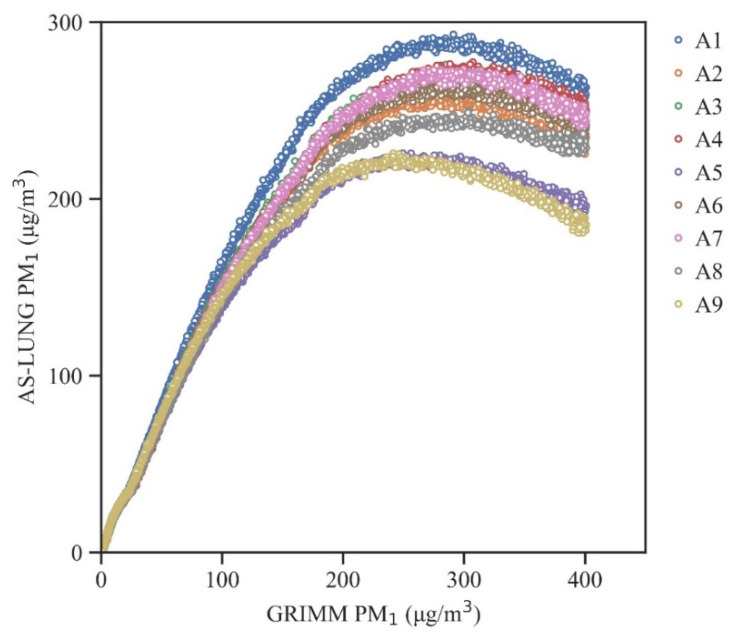
Curves with 0.1–400 µg/m^3^ of PM_1_ for the nine AS-LUNG-P sets.

For PM_1_, the correction curves turned downward above 200 µg/m^3^ (Figure 5), indicating possible saturation of the sensors. Different sensors from the same batch turned downward at different points. Therefore, 200 µg/m^3^ was taken as the upper limit of PM_1_. The correction equations of PM_1_ chosen for further applications are shown in Table 3b. For PM_2.5_, no ceiling values were observed in our experiments.

### 3.4. Incense Versus Mosquito Coils

In the comparison between the incense and mosquito coils, we found that the linear regressions for the mosquito coils have slightly lower slopes than those of incense (Table 5): 2.65–2.89 for PM_2.5_ and 1.34–1.56 for PM_1_, with an R^2^ of 0.987–0.998. The linear regressions from the chamber experiments with the mosquito coils have similar %CVs in their slopes and RMSEs for PM_2.5_ and PM_1_ compared to those of the incense. The ICC indexes (0.995–0.999) are excellent, indicating good repeatability in these experiments (Table 2a). However, these linear regressions with mosquito coils still have large intercepts for PM_2.5_.

The same as in the case of the incense, PM_1_ turned downward above 200 µg/m^3^. Thus, segmented regressions were applied for PM_2.5_ values up to 400 µg/m^3^ and up to 200 µg/m^3^ for PM_1_; these regressions are shown in Table 6a–d. The equations have excellent R^2^ values (0.999) and small intercepts (less than 2.1), which are similar to those from the incense experiments. For PM_1_, the regressions have intercepts of ± 1, which are even better than those from the incense experiments. They also have low inter-sensor variability, with a %CV less than 11.1% for the slopes and RMSEs for both PM_2.5_ and PM_1_ of 0.1–200 µg/m^3^. The overall RMSEs for PM_2.5_ are 1.08 ± 0.02, 1.36 ± 0.16, and 1.57 ± 0.14 µg/m^3^ for 0.1–200, 0.1–300, and 0.1–400 µg/m^3^, respectively (Table 4). 

These values are similar to those from the incense experiments. The ICCs between the mosquito coil and incense experiments are above 0.995 under both linear regression (Table 2a) and segmented regression for both PM_2.5_ and PM_1_ (not shown in the table), demonstrating that the two test materials are interchangeable. Using mosquito coils obtained similar correction equations to those found with the incense.

### 3.5. Sensor Drift

Whether correction equations will drift over time and how often laboratory re-evaluations and on-site maintenance should be conducted are essential questions for sensor networks. The individual CONC_Pre-Test_ and CONC_Post-Test_ linear regressions were obtained before and after the field campaigns 1.5 years apart with incense in the hood experiments. The R^2^ values for the three sets were all fairly good (R^2^ = 0.83–0.99). In terms of drifting, for the two sets that were cleaned on-site, the slopes of both tests were close to each other. The absolute percentage differences of these two sets between the CONC_Pre-Test_ and CONC_Post-Test_ were 24% and 19%, respectively. In other words, the sensor response drifted roughly 20% over 1.5 years. It should be emphasized that these two sets in the field encountered similar PM_2.5_ levels with mean PM_2.5_ values of 16.1 and 19.3 µg/m^3^ in July and 31.1 and 33.1 µg/m^3^ in December. Moreover, the PM_2.5_ levels were 17.8 µg/m^3^ in July and 34.1 µg/m^3^ in December, which are close to the PM_2.5_ levels of the two sets with cleaning (for the other set without on-site cleaning). The correction equation drifted significantly, with 110% for the absolute percentage difference of the other set without cleaning. This evaluation demonstrates that thorough cleaning should be conducted to maintain good data quality. If 15% drift is acceptable, an annual evaluation is required to maintain good data quality, based on 19–24% drift over 1.5 years.

## 4. Discussion

### 4.1. Performance of PMS3003 and AS-LUNG Sets

Air pollution sensor networks are good compliments to current regulatory monitoring networks for providing pollutant levels close to citizens’ living environments in large areas at much lower cost [8,37]. We propose a hybrid method of combining laboratory evaluations and data science to ensure that LCS networks provide accurate PM data. First, LCS data are corrected by laboratory side-by-side comparisons for “seed” LCS devices, which can be installed strategically in areas without EPA stations; secondly, statistical or machine learning methods are applied to adjust nearby uncalibrated LCS devices with data from the EPA or the seed LCS devices wherever available. Thus, readings from other uncalibrated LCS devices in the sensor network can be corrected to nearly research-grade observations accordingly. The current work focuses on the first part of this process to obtain reliable and robust correction equations to convert the readings of LCS devices to research-grade (or FEM-comparable) measurements via side-by-side comparisons with research-grade instruments in the laboratory. The robustness and variability of the acquired correction equations under different experimental settings were evaluated with low-cost considerations.

Our results show that in both the hood and chamber experiments, the AS-LUNG sets with PMS3003 have good agreement with the FEM EDM-180, providing a high R^2^ of 0.930–0.998 in the hood and chamber experiments with linear regressions and 0.999 with segmented regressions, showing that the AS-LUNG sets meet the USEPA’s criteria for continuous PM_2.5_ monitors (r > 0.9 or R^2^ > 0.81) [30] and for candidate equivalent methods (r > 0.97 or R^2^ > 0.94) [31]. However, the slopes of these regression lines do not meet the USEPA’s criteria (1 ± 1) [30]. This accuracy issue could be solved via the presented laboratory evaluation methodologies. With side-by-side comparisons, both the AS-LUNG-P and AS-LUNG-O readings could be converted to EDM-180-comparable measurements and serve as “seed” LCS devices in sensor networks. PMS3003 is not the newest Plantower sensor on the market; however, for research purposes, a sensor with a high R^2^ with FEM instruments is much better to provide reliable data than the fancy ones with unknown drawbacks.

Our results also show that chamber experiments with better seals can acquire correction equations with a much lower variability between different LCS devices and duplicate experiments for PM_2.5_ and PM_1_ (higher ICC indexes) than the hood experiments. Since more observations at higher concentrations typically ensure the robustness of the regression equations, correction equations from the chamber are taken as more accurate estimations than the hood corrections. Without correction, the PMS3003 readings can overestimate PM_2.5_ by about 2–3 fold and PM_1_ by about 1.4–2.2 fold. It should be noted that PMS3003 seems to have an upper limit of around 200–250 µg/m^3^ for PM_1_ but can detect PM_2.5_ up to 400 µg/m^3^.

Both the hood and chamber experiments were able to obtain correction equations with high R^2^ values and high ICC indexes (0.952–0.999), showing the excellent precision of AS-LUNG and the excellent repeatability of the presented experimental settings and protocol. The choice of experimental settings needs to consider the required expenses and acceptable degrees of variability. The advantage of using a chemical fume hood is that a hood is a standard set-up in a wet laboratory and does not require extra costs compared to a chamber. Using incense in hoods for side-by-side comparisons would encounter large variability with 10.9–15.5% for slopes with a mean RMSE of 1.67–2.40 for PM_2.5_ and 7.6–10.1% for slopes with mean RMSEs of 1.38–1.82 for PM_1_ (from both AS-LUNG-P and AS-LUNG-O). On the other hand, if resources permit, a chamber is a better choice for conducting evaluation experiments to acquire correction equations. With segmented regressions, the mean RMSEs of PM_2.5_ are less than 1.18 µg/m^3^ with %CVs less than 6.0% for the slopes and RMSEs in the range of 0.1–200 µg/m^3^ with both incense and mosquito coils in the chamber experiments, with a slight increase in the %CV and RMSEs as the concentration increases. For PM_1_ of 0.1–200 µg/m^3^, the mean RMSEs are less than 1.56–1.63 µg/m^3^, with inter-sensor variability of less than 11.8% with either incense or mosquito coils. These results demonstrate the steadiness of the PMS3003 sensor. Higher expenses provide better sealed conditions in chamber experiments with a reduced %CV. Our %CV results in the chamber experiments for PM_2.5_ were within the USEPA’s acceptable measurement uncertainty for continuous PM_2.5_ monitors (%CV < 10%) [30].

Performance evaluations for PMS3003 have also been reported by other research groups. PMS3003 was assessed in wind tunnels, and high correlations were found with GRIMM 1.109 (R^2^ = 0.73–0.97), with linearity of 200–850 µg/m^3^ [23]. Another chamber evaluation of data from 242 PMS3003 sensors found high linear correlations (R^2^ > 0.978) with a DustTrak monitor with ammonium nitrate and alumina oxide, providing small intercepts, good repeatability, and certain deviations from the reference values [20], similar to most of the results presented in this work. Additionally, the authors found significant differences between the responses of the sensors purchased from different batches, indicating the necessity to calibrate each batch. Moreover, field evaluations were conducted for PMS3003 sensors in two suburban regions with a mean 1 h PM_2.5_ of 9 ± 9 and 10 ± 3 µg/m^3^ and, in one location, a 1 h PM_2.5_ of 36 ± 17 and 116 ± 57 µg/m^3^ during the monsoon and post-monsoon seasons, respectively [24]. These results showed excellent intra-PMS3003 precision (R^2^ = 0.98–1.00), but their correlations with the reference instruments were not good. An RMSE of 3 µg/m^3^ was found, with a quadratic fit for a 24 h integration time against an E-BAM, presenting non-linearity at high-levels above 300 µg/m^3^ [24]. Our work showed a high R^2^ (0.930–0.999) with a breakpoint around 30–40 µg/m^3^ in the range of 0.1–400 µg/m^3^ for PM_2.5_ (the highest level generated in our experiments) and a non-linear response above 200 µg/m^3^ for PM_1_.

Moreover, two other PMS sensor models, a PMS1003 and a PMS5003, were evaluated in Utah (USA), for 320 days against TEOM, and their RMSE values were found to be above 10 µg/m^3^—much higher than our results in the laboratory [25]. In addition, 19 AirBeams were compared against a BAM in California (USA), with a mean RMSE of 1.08 µg/m^3^ [38]. Our evaluation in the laboratory showed that the RMSE performance of PMS3003 is better than, or at least comparable to, that of other evaluated sensors.

The signal drift of the sensors after the 1.5 years field campaign was shown to be only 19–24%. Side-by-side comparisons may thus be needed once a year to maintain the validity of the correction equations. In addition, employing a cleaning procedure is also required to maintain good data quality of the sensors. Whether the sensor drift changed linearly over time or occurred suddenly needs to be further evaluated. It was found that the signals of PMS1003 did not change after one year of field operations in the USA [25]. Another sensor, the AirBeam, drifted less than 5% before and after a two-month campaign in the USA [38]. LCS devices have received significant attention for their potential applications. However, the potential drift of sensor responses and the required maintenance of such sensors have not been documented prior to this manuscript. These results demonstrate that wherever data accuracy is important for long-term monitoring, proper maintenance is mandatory. More works need to be done to better assess sensor drift.

### 4.2. Choices of Evaluation Settings

Traditionally, aerosol scientists have tended to use expensive standard dust, such as Arizona road dust or urban dust, to evaluate sensor performance. To maintain our low-cost principle, we used commercially available and inexpensive aerosols for our evaluations. To avoid a fire hazard, incense sticks and mosquito coils were chosen as our test materials rather than straw, and cigarettes were not considered due to their tar contents, which could contaminate the chamber surfaces. Although the ingredients of the incense sticks and mosquito coils differ greatly, the acquired correction equations are quite similar, implying the robustness of the correction equations. Incense sticks can be purchased worldwide for traditional, religious, or relaxation purposes. Thus, incense sticks are recommended to be used as economic burnt material examples for side-by-side comparisons. Additionally, for sensor evaluations, previous work has shown that incense offers similar performance to PM_2.5_ in residential air in Baltimore, suggesting that incense may be a suitable substitute for urban PM_2.5_ [22]. The results of the current work and our previous work [10] also support the use of incense sticks, representing urban PM_2.5_, for the evaluation of LCS.

In this work, segmented regression was applied to obtain the correction equations for 0.1–200, 0.1–300, 0.1–400 µg/m^3^ of PM_2.5_ from the chamber experiments. These correction equations have much smaller intercepts than those of the linear regressions from either the hood or chamber experiments, with much smaller RMSEs. The R^2^ values are 0.999 for all three concentration ranges. Therefore, segmented regressions are recommended for the correction equations, rather than linear regressions.

Moreover, our side-by-side comparisons were conducted with two GRIMM 1.109 instruments. Based on their good agreement with the EDM-180, a FEM instrument, the final correction equations were constructed to convert AS-LUNG readings into FEM-comparable values. The GRIMM device is smaller and easier to carry around. If only research-grade measurements are needed, correction experiments with the GRIMM 1.109 are sufficient. However, if FEM comparable measurements are preferred and resources permit, EDM-180 is recommended as an optimal instrument to use for side-by-side comparisons.

For scientists who have the resources to conduct laboratory evaluations, this work provides valuable information on the choice of experimental setting (i.e., a chemical fume hood versus chamber), the materials used (incense versus mosquito coils), and linear or segmented regression equations. Different correction equations were compared in this study to illustrate possible biases and variability under different experimental conditions. Traditional methods of conducting side-by-side comparisons with research instruments typically use standard dust and required repeated experiments under pre-specified temperatures and RH conditions inside a temperature and RH controlled chamber, thus requiring more resources. The greatest advantage of our method is that one can acquire robust correction equations for a stable PM sensor to obtain FEM-comparable data with considerably lower costs and result that are closer to real-world scenarios than those obtained using traditional methods.

For scientists who have only limited resources and intend to use AS-LUNG sets in areas of interest with PM_2.5_ concentrations higher than 10 µg/m^3^, hood experiments with incense and GRIMM 1.109 with linear regressions are sufficient. Lower costs come with higher variability in the slopes and RMSEs. The large intercepts of correction equations would not be an issue in polluted areas. Even with 15–20% variability, AS-LUNG sets, after conversion, can be used as seed LCS devices for the reading adjustment of other uncalibrated LCS devices. The raw measurements with 2–3 times overestimations can be corrected to a more acceptable concentration range.

Due to economic considerations, only one experiment under certain temperature and RH conditions is preferred to acquire one correction equation for one LCS device. The conditions for one experiment cannot cover the wide range of all environmental conditions in the field. Extra side-by-side comparisons should be carried out during different seasons in the field to obtain correction equations covering wider temperature and humidity ranges. This process should be less expensive than setting up comparisons in a temperature- and humidity-controlled chamber. The correction equations established in this work are intended to be applied in the field in subtropical Taiwan, under a temperature of 15–30 °C and humidity of 70–84% based on the monthly means in non-mountainous areas from 1981–2010 [39]. Certain subtropical areas, such as southeastern Asia, have similar climatic conditions to Taiwan with high PM levels. This inexpensive method of conducting side-by-side evaluations could also be carried out in these countries to facilitate the development of LCS networks.

A limitation of this work is that the provided correction equations may not be applicable in other countries with different temperatures and humidity ranges, although this methodology still has practical value. The impacts of temperature and humidity on PM LCS have also been evaluated by different research groups [20,24,31,40]. Two groups, for example, developed correction equations that consider temperature and humidity [24,40]. While our group did not adjust for temperature and humidity based on the aforementioned reasons and due to the subtropical climatic conditions in Taiwan, we acknowledge the need to acquire correction equations under different temperature and humidity ranges for other research groups. For countries located in different climate zones, one or two more correction equations at lower/higher temperatures and dried humidity may be required for the equations to be applicable in the field.

## 5. Conclusions

In conjunction with machine learning methods, traditional laboratory evaluations could be applied for limited sets of seed LCS devices installed in areas of interest without official monitoring stations for the adjustment of other uncalibrated LCS devices in the sensor networks and enhance the data quality and potential applications of the sensor networks. This work provides methodologies to acquire robust correction equations to obtain FEM-comparable data from LCS devices at much lower costs. Our results demonstrate that two LCS devices with PMS3003 sensors, AS-LUNG-P and AS-LUNG-O, are good choices for seed LCS devices. The correction equations obtained inside the chamber with segmented regressions had a high R^2^ of 0.999, less than 6.0% variability in their slopes, and mean RMSEs of 1.08–1.18 µg/m^3^ for 0.1–200 µg/m^3^ of PM_2.5_ with either incense or mosquito coils. For PM_1_ in the range of 0.1–200 µg/m^3^, the correction equations had an R^2^ of 0.999, less than 11.8% variability in their slopes, and mean RMSEs of 1.49–1.63 µg/m^3^ using either incense or mosquito coils. For scientists with only limited resources, this evaluation can be conducted inside a typical chemical fume hood, under which correction equations can be obtained with an R^2^ of 0.930 to 0.996, 7.6–15.5% variability in their slopes, and mean RMSEs of 1.67–2.40 and 1.38–1.82 µg/m^3^ for PM_2.5_ and PM_1_, respectively. Substantial resources are needed to maintain one air-conditioned monitoring station with expensive instruments. In comparison, maintaining the seed LCS sensor network for the reading adjustment of other LCS devices requires fewer resources, which can provide FEM-comparable observations over large areas. Interested research groups or regulatory agencies could follow our suggestions to choose a proper way to establish a bias correction equation that can be incorporated into their sensor networks.

## Figures and Tables

**Table 1:** Linear correction equations from the hood and chamber experiments with incense for (**a**) PM_2.5_ with nine AS-LUNG-P sets, (**b**) PM_1_ with 9 AS-LUNG-P sets, and (**c**) PM_2.5_ and PM_1_ with 12 AS-LUNG-O sets in the range of 0.1–200 μg/m^3^.

**(a) PM_2.5_**	**Hood with Incense**					**Chamber with Incense**				
	**T^1^: 24.7–28.6 °C, RH: 57.3–76.0%**					**T: 28.0–29.9 °C, RH: 36.7–40.2%**				
	**Slope**	**Intercept**	**R^2^**	**RMSE^2^**	***n***	**Slope**	**Intercept**	**R^2^**	**RMSE**	***n***
A1	3.11	−9.02	0.981	1.40	840	3.10	−15.4	0.993	4.76	1733
A2	3.19	−6.96	0.991	0.95	840	2.92	−10.8	0.996	3.56	1733
A3	3.39	−10.1	0.994	0.78	840	2.92	−12.5	0.995	4.08	1733
A4	3.59	−7.26	0.991	1.26	827	2.96	−13.1	0.994	4.27	1733
A5	2.44	−6.20	0.930	3.89	665	2.93	−9.80	0.997	3.25	1733
A6	3.52	−6.82	0.989	1.42	827	2.94	−12.7	0.994	4.11	1732
A7	2.71	−4.67	0.976	2.07	826	2.94	−13.4	0.994	4.38	1732
A8	2.51	−3.24	0.985	1.63	829	2.85	−10.6	0.996	3.54	1733
A9	2.49	−2.49	0.985	1.61	829	2.80	−9.11	0.996	3.30	1732
Average	2.99	−6.30	0.980	1.67		2.927	−11.9	0.995	3.92	
SD	0.47	2.49	0.020	0.92		0.083	1.98	0.001	0.53	
%CV^3^	15.5%	−39.6%		54.9%		2.8%	−16.6%		13.4%	
**(b) PM_1_**	**Hood with Incense**					**Chamber with Incense**				
	**T: 24.7–28.6 °C, RH: 57.3–76.0%**					**T: 28.0–29.9 °C, RH: 36.7–40.2%**				
	**Slope**	**Intercept**	**R^2^**	**RMSE**	***n***	**Slope**	**Intercept**	**R^2^**	**RMSE**	***n***
A1	1.88	−3.23	0.991	0.95	840	1.69	−1.4	0.997	2.92	1733
A2	1.90	−0.96	0.990	1.03	840	1.60	1.2	0.995	3.93	1733
A3	2.00	−3.3	0.986	1.21	840	1.62	−0.3	0.997	3.11	1733
A4	2.24	−3.72	0.988	1.47	827	1.68	−1.4	0.998	2.66	1733
A5	2.13	−5.53	0.945	3.45	665	1.48	3.40	0.991	5.15	1733
A6	2.15	−3.09	0.988	1.45	827	1.59	0.1	0.997	3.24	1732
A7	1.81	−2.71	0.994	0.99	826	1.61	−1.1	0.997	2.81	1732
A8	1.79	−2.02	0.994	1.04	829	1.59	0.7	0.996	3.50	1733
A9	1.65	−1.19	0.996	0.87	829	1.40	3.51	0.991	5.26	1732
Average	1.95	−2.86	0.986	1.38		1.58	0.53	0.995	3.62	
SD	0.20	1.39	0.016	0.80		0.091	1.87	0.003	0.98	
%CV	10.1%	−48.5%		58.0%		5.7%	356.4%		26.9%	
**(c) PM**	**Hood with Incense (PM_2.5_)**					**Hood with Incense (PM_1)_**				
	**T: 20.8–34.1 °C, RH: 30.4–64.1%**					**T: 20.8–34.1 °C, RH: 30.4–64.1%**				
	**Slope**	**Intercept**	**R^2^**	**RMSE**	***n***	**Slope**	**Intercept**	**R^2^**	**RMSE**	***n***
B1	2.27	−4.32	0.986	1.66	768	1.71	−4.41	0.993	1.19	768
B2	2.28	−3.17	0.986	1.58	819	1.69	−3.14	0.994	1.02	819
B3	2.15	−2.14	0.968	2.44	785	1.73	−4.31	0.979	2.01	785
B4	2.04	−3.73	0.982	1.85	785	1.51	−3.30	0.992	1.25	785
B5	2.55	−6.72	0.979	2.82	417	1.77	−3.59	0.987	2.21	417
B6	2.43	−7.97	0.982	2.59	419	1.69	−4.39	0.987	2.17	419
B7	2.43	−6.46	0.975	2.94	407	1.73	−5.66	0.984	2.33	407
B8	2.36	−4.68	0.985	2.33	772	1.67	−2.65	0.984	2.35	772
B9	2.08	−6.57	0.969	2.47	638	1.69	−4.86	0.983	1.85	638
B10	2.37	−6.93	0.984	2.34	620	1.81	−4.30	0.992	1.59	620
B11	2.90	−10.39	0.979	2.31	829	1.95	−4.87	0.992	1.38	829
B12	2.64	−9.22	0.985	1.93	821	1.91	−4.85	0.994	1.26	821
Average	2.42	−6.96	0.980	2.40		1.75	−4.27	0.988	1.82	
SD	0.26	2.06	0.005	0.36		0.13	0.94	0.004	0.46	
%CV	10.9%	−29.6%		15.1%		7.6%	−21.9%		25.3%	

^1^ Temperature and humidity range of the first experiment and the duplicate run. ^2^ RMSE: root mean square error. ^3^ Inter-sensor variability was assessed with the percent coefficients of variance (%CV = standard deviation/mean) of the results.

**Table 2:** Intra-class correlation coefficient (ICC) from the hood and chamber experiments with duplicates for (**a**) PM_2.5_ and PM_1_ with 9 AS-LUNG-P sets with incense and mosquito coils and (**b**) PM_2.5_ and PM_1_ with 12 AS-LUNG-O sets with incense in the range of 0.1–200 μg/m^3^.

**(a)**	**Hood with Incense**		**Chamber with Incense**		**Chamber with Mosquito Coils**	
	**PM_2.5_**	**PM_1_**	**PM_2.5_**	**PM_1_**	**PM_2.5_**	**PM_1_**
A1	0.969	0.993	0.997	0.999	0.997	0.998
A2	0.970	0.995	0.998	0.999	0.998	0.997
A3	0.952	0.988	0.998	0.999	0.998	0.998
A4	0.928	0.970	0.998	1.000	0.997	0.997
A5	0.994	0.980	0.999	0.999	0.998	0.996
A6	0.965	0.988	0.998	0.999	0.998	0.997
A7	0.982	0.985	0.998	0.999	0.998	0.997
A8	0.979	0.981	0.999	0.999	0.998	0.997
A9	0.975	0.980	0.999	0.997	0.999	0.995
Average	0.968	0.984	0.998	0.999	0.998	0.997
SD	0.019	0.008	0.001	0.001	0.001	0.001
	**(b) Hood with Incense**					
	**PM_2.5_**			**PM_1_**		
B1	0.995			0.995		
B2	0.997			0.998		
B3	0.991			0.996		
B4	0.993			0.991		
B5	0.996			0.998		
B6	0.988			0.981		
B7	0.993			0.985		
B8	0.997			0.997		
B9	0.992			0.998		
B10	0.998			0.992		
B11	0.995			0.987		
B12	0.988			0.972		
Average	0.993			0.989		
SD	0.004			0.009		

**Table 3:** Segmented regression equations from the chamber experiments with incense for (**a**) 0.1–200 μg/m^3^ of PM_2.5_, (**b**) 0.1–200 μg/m^3^ of PM_1_, (**c**) 0.1–300 μg/m^3^ of PM_2.5_, and (**d**) 0.1–400 μg/m^3^ of PM_2.5_.

**(a) PM_2.5_**	**Chamber with Incense (0.1–200 μg/m^3^) with Segmented Regressions**									
	**T: 27.5–30.7 °C, RH: 47.7–54.1%**									
	**Region 1**				**Region 2**					
	**Slope 1**	**Intercept 1**	**BP^1^ 1**		**Slope 2**	**Intercept 2**		**R^2^**		***n***
A1	2.48	2.21	31.7		3.06	−16.2		0.999		1909
A2	2.44	2.18	31.8		2.79	−9.1		0.999		1905
A3	2.40	2.12	33.9		2.80	−11.5		0.999		1887
A4	2.28	2.06	36.0		2.72	−13.7		0.999		1873
A5	2.47	2.16	29.6		2.75	−6.2		0.999		1913
A6	2.39	2.24	31.9		2.84	−12.0		0.999		1895
A7	2.38	2.12	34.7		2.86	−14.5		0.999		1873
A8	2.35	2.20	31.7		2.70	−8.9		0.999		1877
A9	2.70	0.46	151.7		2.44	40.2		0.999		1915
Average	2.43	1.97			2.77			0.999		
SD	0.12	0.57			0.16			0.000		
%CV	4.8%	28.9%			5.9%					
**(b) PM_1_**	**Chamber with Incense (0.1–200 μg/m^3^) with Segmented Regressions**									
	**T: 27.5–30.7 °C, RH: 47.7–54.1%**									
	**Region 1**				**Region 2**					
	**Slope 1**	**Intercept 1**	**BP 1**		**Slope 2**	**Intercept 2**		**R^2^**		***n***
A1	1.64	1.28	107.8		1.03	66.8		0.999		1909
A2	1.51	1.70	103.1		0.89	65.3		0.999		1905
A3	1.53	1.52	104.1		0.96	60.2		0.999		1887
A4	1.47	1.21	110.3		0.97	56.3		0.999		1873
A5	1.43	2.06	97.7		0.75	67.8		0.999		1913
A6	1.52	1.64	103.1		0.93	63.0		0.999		1895
A7	1.49	1.28	105.8		0.99	54.5		0.999		1873
A8	1.45	1.71	101.7		0.84	63.5		0.999		1877
A9	1.50	2.24	95.2		0.72	76.5		0.999		1915
Average	1.50	1.63			0.90			0.999		
SD	0.061	0.35			0.11			0.000		
%CV	4.0%	21.8%			11.8%					
**(c) PM_2.5_**	**Chamber with Incense (0.1–300 μg/m^3^) with Segmented Regressions**									
	**T: 27.5–30.7 °C, RH: 47.7–54.1%**									
	**Region 1**				**Region 2**					
	**Slope 1**	**Intercept 1**	**BP 1**		**Slope 2**	**Intercept 2**		**R^2^**		***n***
A1	2.50	2.11	28.8		3.01	−12.7		0.999		2113
A2	2.72	−1.18	184.7		2.60	22.0		0.999		2109
A3	2.43	1.95	29.2		2.74	−7.2		0.999		2091
A4	2.30	1.94	31.6		2.67	−9.7		0.999		2077
A5	2.71	−0.51	170.7		2.55	26.2		0.999		2117
A6	2.43	2.04	28.2		2.78	−8.0		0.999		2099
A7	2.40	2.02	30.7		2.81	−10.7		0.999		2077
A8	2.63	−1.20	182.8		2.50	23.3		0.999		2081
A9	2.70	0.47	145.2		2.50	29.6		0.999		2119
Average	2.53	0.85			2.68			0.999		
SD	0.16	1.46			0.17			0.000		
%CV	6.2%	172.1%			6.3%					
**(d) PM_2.5_**	**Chamber with Incense (0.1–400 μg/m^3^) with Segmented Regressions**									
	**T: 27.1–30.7 °C, RH: 47.7–54.7%**									
	**Region 1**			**Region 2**			**Region 3**			
	**Slope 1**	**Intercept 1**	**BP 1**	**Slope 2**	**Intercept 2**	**BP 2**	**Slope 3**	**Intercept 3**	**R^2^**	***n***
A1	2.48	2.17	30.1	3.03	−14.2	342.6	5.08	−716.6	0.999	2359
A2	2.69	−0.30	332.2	3.45	−251.5	377.7	5.36	−971.7	0.999	2355
A3	2.41	2.02	30.3	2.75	−8.19	349.9	4.78	−717.7	0.999	2337
A4	2.30	1.98	32.5	2.68	−10.5	351.3	4.60	−684.2	0.999	2323
A5	2.71	−0.49	150.8	2.60	14.8	348.6	4.11	−508.8	0.999	2363
A6	2.42	2.11	29.2	2.80	−8.96	347.4	4.86	−726.9	0.999	2345
A7	2.40	2.05	31.6	2.82	−11.4	348.1	4.59	−627.7	0.999	2323
A8	2.43	1.74	26.2	2.62	−3.02	355.3	4.05	−510.7	0.999	2327
A9	2.70	0.51	131.1	2.55	19.7	336.4	4.18	−528.3	0.999	2365
Average	2.50	1.31		2.81			4.62		0.999	
SD	0.15	1.09		0.28			0.45		0.000	
%CV	6.2%	83.3%		9.9%			9.8%			

^1^ BP: break point.

**Table 4:** Root mean square errors (RMSEs) of different regions based on the segmented regression equations from the chamber experiments with incense and mosquito coils for (**a**) 0.1–200 μg/m^3^ of PM_2.5_, (**b**) 0.1–200 μg/m^3^ of PM_1_, (**c**) 0.1–300 μg/m^3^ of PM_2.5_, and (**d**) 0.1–400 μg/m^3^ of PM_2.5_.

**(a) PM_2.5_**	**Chamber with Incense (0.1–200 μg/m^3^)**			**Chamber with Mosquito Coils (0.1–200 μg/m^3^)**				
	**Region 1**	**Region 2**	**Overall**	**Region 1**	**Region 2**		**Overall**	
A1	1.08	1.21	1.13	1.11	1.00		1.07	
A2	1.11	1.25	1.16	1.09	1.03		1.07	
A3	1.11	1.19	1.14	1.08	1.00		1.05	
A4	1.15	1.18	1.16	1.13	0.98		1.08	
A5	1.09	1.42	1.22	1.06	1.18		1.10	
A6	1.13	1.20	1.15	1.13	0.97		1.07	
A7	1.12	1.06	1.09	1.13	1.01		1.09	
A8	1.15	1.29	1.20	1.14	0.98		1.08	
A9	1.31	1.64	1.33	1.11	1.14		1.12	
Average	1.14	1.27	1.18	1.11	1.03		1.08	
SD	0.07	0.17	0.07	0.03	0.08		0.02	
%CV			6.0%				1.9%	
**(b) PM_1_**	**Chamber with Incense (0.1–200 μg/m^3^)**			**Chamber with Mosquito Coils (0.1–200 μg/m^3^)**				
	**Region 1**	**Region 2**	**Overall**	**Region 1**	**Region 2**		**Overall**	
A1	1.21	2.83	1.51	1.62	2.53		1.73	
A2	1.27	2.52	1.51	1.47	2.05		1.56	
A3	1.24	2.25	1.42	1.50	1.89		1.55	
A4	1.30	2.13	1.41	1.67	1.97		1.71	
A5	1.36	3.16	1.77	1.32	2.79		1.62	
A6	1.28	2.59	1.53	1.44	2.46		1.60	
A7	1.27	2.32	1.45	1.63	2.32		1.72	
A8	1.33	2.45	1.55	1.41	2.16		1.53	
A9	1.34	3.41	1.85	1.28	2.94		1.64	
Average	1.29	2.63	1.56	1.48	2.35		1.63	
SD	0.05	0.43	0.15	0.14	0.37		0.08	
%CV			9.9%				4.7%	
**(c) PM_2.5_**	**Chamber with Incense (0.1–300 μg/m^3^)**			**Chamber with Mosquito Coils (0.1–300 μg/m^3^)**				
	**Region 1**	**Region 2**	**Overall**	**Region 1**	**Region 2**		**Overall**	
A1	1.08	1.68	1.38	1.11	1.31		1.19	
A2	1.56	2.17	1.64	1.11	1.77		1.45	
A3	1.11	1.83	1.48	1.09	1.36		1.22	
A4	1.15	1.69	1.41	1.13	1.41		1.26	
A5	1.44	2.26	1.57	1.06	2.09		1.64	
A6	1.11	1.80	1.46	1.13	1.37		1.24	
A7	1.12	1.61	1.36	1.13	1.44		1.28	
A8	1.61	2.13	1.68	1.15	1.87		1.53	
A9	1.31	2.04	1.45	1.12	1.63		1.39	
Average	1.28	1.91	1.49	1.12	1.58		1.36	
SD	0.21	0.24	0.11	0.03	0.27		0.16	
%CV			7.6%				11.6%	
**(d) PM_2.5_**	**Chamber with Incense (0.1–400 μg/m^3^)**			**Chamber with Mosquito Coils (0.1–400 μg/m^3^)**				
	**Region 1**	**Region 2**	**Region 3**	**Overall**	**Region 1**	**Region 2**	**Region 3**	**Overall**
A1	1.08	1.93	3.31	1.71	1.11	1.34	4.31	1.44
A2	1.83	2.46	2.45	1.88	1.11	1.63	4.17	1.63
A3	1.11	2.04	2.96	1.73	1.09	1.37	4.73	1.52
A4	1.15	1.94	3.15	1.70	1.13	1.34	4.01	1.49
A5	1.44	2.57	3.55	1.88	1.06	1.98	5.18	1.92
A6	1.12	2.00	3.10	1.73	1.13	1.37	4.24	1.50
A7	1.12	1.83	3.06	1.64	1.13	1.36	4.11	1.50
A8	1.25	2.21	3.13	1.90	1.15	1.53	3.92	1.63
A9	1.31	2.43	3.31	1.79	1.12	1.53	3.67	1.54
Average	1.27	2.16	3.11	1.77	1.11	1.50	4.26	1.57
SD	0.24	0.27	0.30	0.09	0.03	0.21	0.45	0.14
%CV				5.3%				9.0%

**Table 5:** Linear correction Equations from the chamber experiments using mosquito coils with 9 AS-LUNG-P sets for PM_2.5_ and PM_1_ in a range of 0.1–200 μg/m^3^.

	Chamber with Mosquito Coils (PM_2.5_)					Chamber with Mosquito Coils (PM_1_)				
	T: 28.0–31.4 °C, RH: 60.9–66.0%					T: 28.0–31.4 °C, RH: 60.9–66.0%				
	Slope	Intercept	R^2^	RMSE	*n*	Slope	Intercept	R^2^	RMSE	*n*
A1	2.89	−9.20	0.995	3.54	1831	1.56	0.87	0.997	3.10	1831
A2	2.72	−6.41	0.997	2.80	1826	1.45	2.44	0.994	3.97	1826
A3	2.72	−7.19	0.997	3.05	1819	1.47	1.68	0.996	3.30	1819
A4	2.68	−8.00	0.996	3.36	1805	1.46	0.63	0.997	2.81	1805
A5	2.74	−5.05	0.998	2.33	1842	1.35	4.31	0.990	5.32	1842
A6	2.79	−7.36	0.997	3.04	1824	1.48	2.27	0.995	3.71	1824
A7	2.78	−8.18	0.996	3.29	1800	1.47	1.06	0.997	3.04	1800
A8	2.65	−6.01	0.997	2.70	1821	1.40	2.70	0.994	4.08	1821
A9	2.71	−4.01	0.998	2.18	1846	1.34	5.22	0.987	5.96	1846
Average	2.74	−6.82	0.997	2.92		1.44	2.35	0.994	3.92	
SD	0.072	1.63	0.001	0.46		0.069	1.56	0.003	1.08	
%CV	2.6%	−23.9%		15.8%		4.8%	66.3%		27.4%	

**Table 6:** Segmented regression equations from the chamber experiments with mosquito coils for (**a**) 0.1–200 μg/m^3^ of PM_2.5_, (**b**) 0.1–200 μg/m^3^ of PM_1_, (**c**) 0.1–300 μg/m^3^ of PM_2.5_, and (**d**) 0.1–400 μg/m^3^ of PM_2.5_.

**(a) PM_2.5_**		**Chamber with Mosquito Coils (0.1–200 μg/m^3^) with Segmented Regressions**									
		**T: 27.5–30.7 °C, RH: 47.7–54.1%**									
		**Region 1**					**Region 2**				
		**Slope 1**		**Intercept 1**	**BP^1^ 1**		**Slope 2**	**Intercept 2**	**R^2^**	***n***	
A1		2.19		1.35	42.7		3.15	−39.7	0.999	1831	
A2		2.17		1.58	39.6		2.90	−27.3	0.999	1826	
A3		2.14		1.40	41.9		2.92	−31.1	0.999	1819	
A4		2.07		1.31	43.8		2.90	−35.0	0.999	1805	
A5		2.25		1.59	35.6		2.87	−20.6	0.999	1842	
A6		2.16		1.73	39.0		2.99	−30.6	0.999	1824	
A7		2.16		1.37	43.1		3.00	−35.1	0.999	1800	
A8		2.12		1.60	38.4		2.81	−25.0	0.999	1821	
A9		2.25		2.10	34.4		2.83	−17.9	0.999	1846	
Average		2.17		1.56			2.93		0.999		
SD		0.06		0.25			0.10		0.000		
%CV		2.6%		16.0%			3.5%				
**(b) PM_1_**	**Chamber with Mosquito Coils (0.1–200 μg/m^3^) with Segmented Regressions**										
	**T: 28.0–31.4 °C, RH: 60.9–66.0%**										
	**Region 1**				**Region 2**						
	**Slope 1**		**Intercept 1**	**BP 1**	**Slope 2**		**Intercept 2**	**R^2^**		***n***	
A1	1.64		−0.95	133.9	1.13		67.6	0.999		1831	
A2	1.57		−0.30	120.2	1.01		66.4	0.999		1826	
A3	1.56		−0.48	123.2	1.10		56.7	0.999		1819	
A4	1.53		−0.92	130.2	1.14		49.5	0.999		1805	
A5	1.52		0.48	110.7	0.86		73.1	0.999		1842	
A6	1.59		−0.28	121.0	1.06		63.2	0.999		1824	
A7	1.54		−0.69	131.1	1.10		57.6	0.999		1800	
A8	1.53		−0.13	118.0	0.98		64.0	0.999		1821	
A9	1.55		0.77	107.4	0.82		78.3	0.999		1846	
Average	1.56		−0.28		1.02			0.999			
SD	0.037		0.59		0.11			0.000			
%CV	2.4%		−214%		11.1%						
**(c) PM_2.5_**	**Chamber with Mosquito Coils (0.1–300 μg/m^3^) with Segmented Regressions**										
	**T: 28.0–31.6 °C, RH: 60.9–68.9%**										
	**Region 1**				**Region 2**						
	**Slope 1**		**Intercept 1**	**BP 1**	**Slope 2**		**Intercept 2**	**R^2^**		***n***	
A1	2.20		1.27	44.4	3.17		−41.9	0.999		2074	
A2	2.17		1.58	34.9	2.84		−21.7	0.999		2069	
A3	2.14		1.42	40.0	2.89		−28.8	0.999		2062	
A4	2.06		1.36	42.2	2.88		−33.2	0.999		2048	
A5	2.28		1.44	30.3	2.80		−14.5	0.999		2085	
A6	2.15		1.76	37.0	2.96		−28.0	0.999		2067	
A7	2.14		1.46	40.5	2.97		−32.1	0.999		2043	
A8	2.13		1.54	33.5	2.74		−19.2	0.999		2064	
A9	2.26		2.03	31.0	2.78		−14.2	0.999		2089	
Average	2.17		1.54		2.89			0.999			
SD	0.07		0.23		0.13			0.000			
%CV	3.0%		15.0%		4.5%						
**(d) PM_2.5_**	**Chamber with Mosquito coils (0.1–400 μg/m^3^) with Segmented Regressions**										
	**T: 27.0–31.6 °C, RH: 60.9–74.4%**										
	**Region 1**				**Region 2**			**Region 3**			
	**Slope 1**		**Intercept 1**	**BP 1**	**Slope 2**	**Intercept 2**	**BP 2**	**Slope 3**	**Intercept 3**	**R^2^**	***n***
A1	2.20		1.27	44.2	3.17	−41.6	305.4	1.55	452.0	0.999	2163
A2	2.17		1.59	35.6	2.84	−22.4	289.1	1.50	365.2	0.999	2158
A3	2.14		1.42	40.0	2.89	−28.76	301.0	1.37	429.0	0.999	2151
A4	2.07		1.33	42.8	2.89	−33.8	290.3	1.68	316.6	0.999	2137
A5	2.27		1.48	30.8	2.81	−15.1	289.9	1.23	441.8	0.999	2174
A6	2.15		1.76	37.0	2.96	−28.06	299.0	1.40	437.8	0.999	2156
A7	2.15		1.44	41.0	2.98	−32.6	294.5	1.47	412.2	0.999	2132
A8	2.12		1.57	34.9	2.76	−20.80	277.5	1.72	267.1	0.999	2153
A9	2.26		2.04	31.5	2.79	−14.7	288.8	1.57	335.8	0.999	2178
Average	2.17		1.55		2.90			1.50		0.999	
SD	0.06		0.24		0.12			0.15		0.000	
%CV	2.9%		15.3%		4.3%			10.3%			

^1^ BP: break point.
